# A panel of protein kinase high expression is associated with postoperative recurrence in cholangiocarcinoma

**DOI:** 10.1186/s12885-020-6655-4

**Published:** 2020-02-24

**Authors:** Sureerat Padthaisong, Malinee Thanee, Nisana Namwat, Jutarop Phetcharaburanin, Poramate Klanrit, Narong Khuntikeo, Attapol Titapun, Watcharin Loilome

**Affiliations:** 1 Department of Biochemistry, Faculty of Medicine, Khon Kaen University, 123 Mittraparp Road, Muang District, Khon Kaen, 40002 Thailand; 2 Cholangiocarcinoma Screening and Care Program (CASCAP), Khon Kaen University, Khon Kaen, 40002 Thailand; 3 Cholangiocarcinoma Research Institute, Faculty of Medicine, Khon Kaen University, Khon Kaen, 40002 Thailand; 4 Department of Surgery, Faculty of Medicine, Khon Kaen University, Khon Kaen, 40002 Thailand

**Keywords:** Cholangiocarcinoma, Cancer recurrence, Protein kinase, Tumor marker, Prognostic factor

## Abstract

**Background:**

Cancer recurrence is one of the most concerning clinical problems of cholangiocarcinoma (CCA) patients after treatment. However, an identification of predictive factor on *Opisthorchis viverrini* (OV)-associated CCA recurrence is not well elucidated. In the present study, we aimed to investigate the correlation of twelve targeted protein kinases with CCA recurrence.

**Methods:**

Twelve protein kinases, epidermal growth factor receptor (EGFR), human epidermal growth factor receptor 2, 3, 4 (HER2, HER3, HER4), vascular endothelial growth factor receptor 3 (VEGFR3), vascular endothelial growth factor-C (VEGF-C), erythropoietin-producing hepatocellular carcinoma receptor type-A3 (EphA3), EphrinA1, phosphor-serine/threonine kinase 1 (p-Akt1), serine/threonine kinase 1 (Akt1), beta-catenin and protein Wnt5a (Wnt5a) were examined using immunohistochemistry. Pre-operative serum tumor markers, CA19–9 and CEA were also investigated.

**Results:**

Among twelve protein kinases, EGFR, HER4, and EphA3 were associated with tumor recurrence status, recurrence-free survival (RFS) and overall survival (OS). Multivariate cox regression demonstrated that EGFR, HER4, EphA3 or the panel of high expression of these proteins was an independent prognostic factor for tumor recurrence. The combination of high expression of these proteins with a high level of CA19–9 could improve the predictive ability on tumor recurrence. Moreover, the patients were stratified more accurately when analyzed using the combination of high expression of these proteins with primary tumor (T) or lymph node metastasis (N) status.

**Conclusion:**

EGFR, HER4, EphA3 or the panel of high expression of these proteins is an independent prognostic factor for post-operative CCA recurrence.

## Background

Cholangiocarcinoma (CCA) is a malignant tumor of bile duct epithelium with very high incidence in Thailand, particularly in northeastern region, of which *Opisthorchis viverrini* (OV) infection is reported as the major risk factor of CCA development in this area [1]. CCA is usually asymptomatic in early stage and most patients are diagnosed with CCA when the disease becomes advanced, resulting in poor outcome [2]. Moreover, the recurrence after treatment is nowadays very important, because it is a significant problem for many patients with cancer and is involved in poor prognosis of patients [3]. In CCA, the high recurrence rate was reported in many studies [4, 5]. A precious study reported that most CCA patients developed recurrence within 2 years after surgery and the percentage of recurrence accounted for 62.2% [5]. Recently, recurrence rate in mass-forming type of intrahepatic CCA patients was reported with the recurrence rate of 80%. 1-, 2-, and 3-year RFS rate were very low which were 16.2, 5.4, and 2.7%, respectively. However, the association between RFS and clinicopathological data was not significant [4]. Thus, the effective prognostic biomarkers are required to assess outcome of CCA patients as well as the probability of recurrence after treatment.

Nowadays, there are several markers reported as tumor behavior predictors. They can be used for disease management including progression and the relapse indicators of cancer. Serum tumor markers are the well-established markers for monitoring tumor and have been reported to predict tumor recurrence in many types of cancer [6, 7]. However, molecular biomarkers are widely studied because it is not only used for the predicting of tumor progression or recurrence, but can also be employed as drug target for cancer treatment. Our group previously reported the alteration of protein kinase expression in CCA. We found that many protein kinases were upregulated in CCA tissue and cell lines, including receptor tyrosine kinase, the epidermal growth factor receptor (EGFR) family, vascular endothelial growth factor (VEGFR) receptor, erythropoietin-producing hepatocellular carcinoma (Eph) receptor, and also many down-steam kinases such as serine/threonine kinase or protein kinase B (Akt), and Wnt/beta-catenin signaling pathways [8]. The evaluation of EGFR expression was reported in CCA and associated with poor prognosis of CCA patients [9]. Furthermore, our group also reported that high expressions of VEGFR3, EphA3 and their ligands were correlated with CCA metastasis [10]. The role of protein kinase in PI3K/Akt signaling pathway was also studied in CCA. The results showed that high expression of protein in this pathway was mostly involved in the worse clinical outcome of CCA patients. Moreover, targeting of this pathway using NVP-BEZ235 could inhibit tumor growth and metastasis through reduced protein kinase activation [11]. The association of Wnt/beta-catenin signaling pathway with CCA progression was also reported. The result showed the alteration of Wnt proteins was associated with poor prognosis of CCA patients, and inhibition of beta-catenin expression could inhibit CCA cell growth [12].

Large-scale multi-omics have also been employed in many studies in order to understand the carcinogenesis as well as the progression of disease. In 2015, a previous study reported the genomic alteration which characterized biliary tract cancer (BTC) patients. EGFR family genes including *EGFR*, *ERBB2* (*HER2*), *ERBB3* (*HER3*) were the most activating gene in gallbladder cancer while *EPHA2* mutation was found frequently in intrahepatic CCA (iCCA) [13]. *ERBB2* amplification was reported for 3.9–8.5% of CCAs. This was more frequent in fluke-associated CCA which account for 10.4% compared with 2.7% of fluke-negative CCA, resulting in the elevation of *ERBB2* gene expression in fluke-associated CCA compared with fluke-negative cases. In addition, the upregulation of AKT1 and WNT5B was also reported [14]. Single-nucleotide variations (SNVs) and insertion-deletions (indels) were found in *ERBB3* gene in BTCs (5%). This mutation was significantly enriched in extrahepatic CCA (eCCA) [15]. Recently, Nepal et al. reported that the mutation of *ERBB4* gene was also found in intrahepatic CCA (iCCA). In addition, pathway dysregulation in each subgroup of patients was explored. They found that the patients who have KRAS mutation were enriched for immune-related pathways, ErbB and VEGF pathways. On the other hand, WNT pathway was enriched in patients with *TP53* gene mutation [16].

Since protein kinases play an important role in CCA progression and are involved in poor prognosis of CCA patients. In the current study, we hypothesized that the alteration of these protein kinases including EGFR family, VEGFR3 and its ligand, Eph receptor and its ligand, Akt1 and its activated form, Wnt, and beta-catenin may be used as the predicting markers for post-operative CCA recurrence. Therefore, twelve protein kinases were examined using immunohistochemistry and analyzed against CCA recurrence status, recurrence location, recurrence-free survival (RFS) and overall survival (OS).

## Methods

### Patient selection criteria and follow-up

OV-associated cholangiocarcinoma (CCA) patients who underwent surgery at Srinagarind Hospital, Khon Kaen University, Khon Kaen, Thailand between February, 2007 and December, 2016 were retrospectively studied. In order to avoid the effect of neoadjuvant on protein expression, the patients were excluded if they received either radiotherapy or chemotherapy before operation. Tissue samples were obtained from CCA patients and kept in the BioBank of the Cholangiocarcinoma Research Institute. The clinical information was assessed in all CCA patients including sex, age, tumor location, histology, size of primary tumor (T stage), lymph node metastasis status (N stage), distant metastasis status (M stage), and TNM staging. In addition, tumor makers (carbohydrate antigen 19-9; CA19–9 and carcinoembryonic antigen; CEA) were examined in pre-operative serum.

For the recurrence, first year after surgery, all CCA patients were followed-up every 3 months and every 6 months thereafter. Post-operative recurrence was defined in the patients who developed new tumor which confirmed by computed tomography (CT)/magnetic resonance imaging (MRI). The interval between the date of operation until the date of recurrence or until the last of follow-up was defined as recurrence-free survival (RFS) and the interval between the date of operation until the date of death or until the last of follow-up was defined as overall survival (OS). Early recurrence was defined if patients developed the new tumor within 1 year after surgery, while late recurrence was defined if patients developed the new tumor after 1 year. This study was approved by the Human Research Ethics Committees, Khon Kaen University, Thailand (HE611412).

### Antibodies

The antibodies used in this study were as follows: EGFR (1:50; # ab52894), HER3 (1:25; # ab5470), HER4 (1:150; # ab19391), VEGFR3 (1:100; # ab27278), VEGF-C (1:50; # ab135506), Wnt5a (1:100; # ab72583), Beta-Catenin (1:100; # ab32572), p-Akt1 (1:100; # ab32505), Akt1 (1:50; #ab59380) were purchased from Abcam company, UK. HER2 (1:100, #4290) was purchased from Cell Signaling Technology, Inc., USA. EphrinA1 (1:100; # sc-911) and EphA3 (1:100; # sc-920) were purchased from Santa Cruz Biotechnology, USA. Horseradish peroxidase (HRP)-conjugated secondary antibodies (Dako EnVision, USA).

### Immunohistochemical staining (IHC)

A CCA tissue microarray (TMA) was prepared from two independent puncture from each patient and cut into 4 μm for each section. The expression of protein was investigated using IHC. Briefly, the sections were de-paraffinized with xylene and rehydrated with stepwise of 100, 90, 80 and 70% ethanol, respectively. Microwave cooking was used for antigen retrieval for 10 mins. Then tissue sections were incubated with 0.3% hydrogen peroxide followed by 10% skim milk for 30 mins of each in order to inhibited endogenous hydrogen peroxide activity, and nonspecific binding. After washing the sections were incubated with primary antibodies at room temperature for 1 h followed by 4 °C overnight. The excess antibodies were washed for 3 times using phosphate buffered saline (PBS) with 0.1% tween20 followed by PBS for 5 mins of each. The sections were then incubated with HRP-conjugated secondary antibodies for 1 h, and the excess antibodies were also washed using PBS with 0.1% tween20 followed by PBS for 5 mins of each. A 3, 3’diaminobenzidine tetrahydrochloride (DAB) substrate kit (Vector Laboratories, Inc., CA) was used to develop the signal. The tissues were then counterstained using hematoxylin for 2 mins. After washing, the tissue sections were dehydrated with stepwise of 70, 80, 90, 100% ethanol and xylene, respectively. Tissue sections were mounted with permount, and finally observed under light microscopy.

### Immunohistochemical (IHC) scoring

The expression of each protein was scored based on intensity and frequency which is the proportion of positive cells stanning. The intensity of protein expression was classified into four levels including 0 = negative, 1 = weak, 2 = moderate, and 3 = strong stanning. The proportion of positive cells stanning was semi-qualitatively, and classified into negative = 0%, 1 = 1–25%, 2 = 26–50%, and 3 = more than 50% positive stanning. The grading score was calculated by multiplying between intensity and frequency, and the minimum score was 0 while the maximum score was 9. The grading score of each patient was calculated from the average value of two independent punctures. Finally, the median value was calculated from all cases and used as cut-off point. The patients having a grading score lower, equal to or higher than the median was classified as the low or high expression group, respectively. For the proteins which have a median equal to zero, the patients have a grading score equal to zero, being classified as the negative group, while those with a grading score above zero are classified as the positive group.

### Statistical analysis

Statistical Package for the Social Science; SPSS software v.25 was used to analyze data in this study. Chi-square test was used to analyze the correlation between protein kinase expression with recurrence status and clinicopathological characteristics of CCA patients. The difference in IHC score and tumor marker levels on recurrence and recurrence location was analyzed using the Kruskal-Wallis test and Mann-Whitney U-test. Kaplan-Meier (log-rank) analysis was used to analyze RFS and OS. The predictive ability of protein kinases on RFS and OS was analyzed by Cox proportional hazards regression. Statistical significance was considered if *p*-value less than 0.05.

## Results

### Patients characteristics

**Fig. 1:**
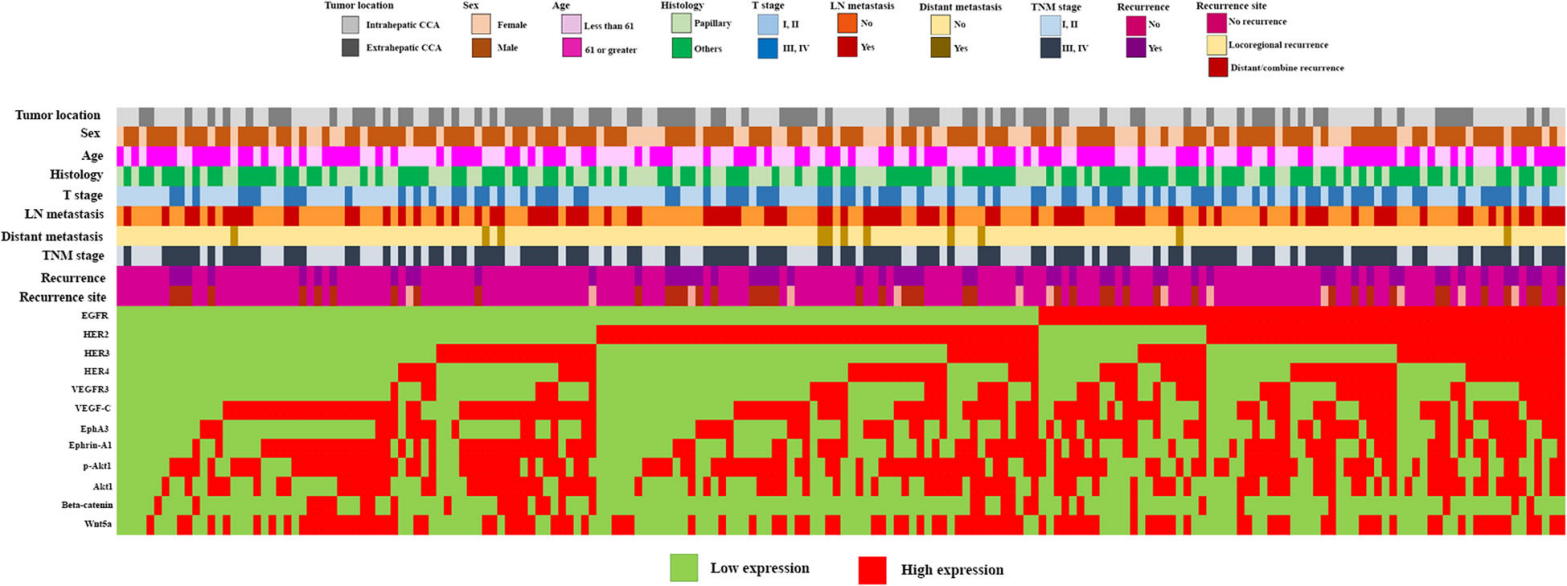
Heatmap showing patients characteristics and expression levels of 190 CCA patients. Top rows indicate clinical characteristics of patients. Bottom rows indicate the expression levels of 12 protein kinases

A total of 190 CCA patients (35% female and 65% male) were recruited in the current study. The median of age was 61 years (rang between 39 and 82). 55% of patients were classified as intrahepatic CCA cases while 45% were extrahepatic CCA cases. 43% of patients were characterized as papillary type and 57% were other types. Size of primary tumor (T) was also classified and 57% of patients were T stage I and II, whereas 43% were T stage III and IV. From 190 patients, lymph node (N) and distant (M) metastasis were shown in 55 and 6% of patients, respectively. TNM staging was also characterized according to size of primary tumor, lymph node and distant metastasis status. In this study, 40% of patients were stage I and II and 60% were stage III and IV and recurrence after surgery was also detected in 31% (Fig. 1) (Table S1). Among patients with recurrence, 53% were classified as early recurrence while 47% were late recurrence. The median follow-up was 16, 28, and 13 months for no recurrence, late recurrence and early recurrence groups, respectively.

### Patterns of recurrence

Among 190 cases, post-operative recurrence was detected in 58 cases (31%) of patients. In this study, pattern of recurrence was divided into locoregional recurrence (22.4%), distant recurrence and combination between locoregional recurrence with distant recurrence (77.6%) (Fig. 1). Locoregional recurrence was defined as a relapse in lymph node (8.6%), anastomosis liver bed (6.9%), and surgical bed (1.7%). In addition, distant recurrence was defined when tumor was detected in other locations including liver (17.2%), lung (5.2%), peritoneum (8.6%), mesentery (1.7%), skin (6.9%), bone (1.7%), and brain (1.7%). Moreover, the multiple recurrences were also detected, and the patterns of recurrence was summarized in Table 1.

**Table 1:** Summary of initial recurrence locations in CCA patients

Recurrence location	Number of CCA patients (%)
Locoregional recurrence	
Surgical bed	1 (1.7%)
Anastomosis liver bed	4 (6.9%)
Lymph node	5 (8.6%)
Lymph node and anastomosis liver bed	3 (5.2%)
Distant recurrence/combination between locoregional recurrence with distant recurrence	
Liver	10 (17.2%)
Lung	3 (5.2%)
Peritoneum	5 (8.6%)
Mesentery	1 (1.7%)
Skin	4 (6.9%)
Bone	1 (1.7%)
Brain	1 (1.7%)
Liver and lymph node	5 (8.6%)
Liver and anastomosis liver bed	1 (1.7%)
Lung and lymph node	1 (1.7%)
Lung and peritoneum and lymph node and bone	1 (1.7%)
Liver and peritoneum	2 (3.4%)
Lung and liver and lymph node	1 (1.7%)
Peritoneum and lymph node	2 (3.4%)
Liver and anastomosis liver bed and peritoneum	1 (1.7%)
Lung and anastomosis liver	1 (1.7%)
Lung and surgical bed	1 (1.7%)
Liver and peritoneum and lymph node	1 (1.7%)
Lung and liver and peritoneum	1 (1.7%)
Liver and skin	1 (1.7%)
Peritoneum and surgical bed	1 (1.7%)
Total	**58 (100%)**

### The correlation of protein kinases with post-operative recurrence and clinicopathological characteristics

**Fig. 2:**
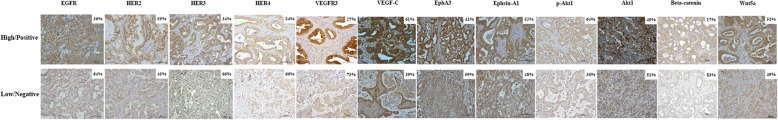
The representative figures of IHC staining, ×200 and the percentages of high/positive and low/negative expression. High/positive and low/negative expression of protein kinases were shown in the upper and lower panel, respectively. The percentages of high/positive and low/negative expression were shown in the white boxes

In the present study, 12 protein kinases including EGFR, HER2, HER3, HER4, VEGFR3, VEGF-C, EphA3, EphrinA1, p-Akt1, Akt1, beta-catenin and Wnt5a were examined in CCA tissues obtained from190 cases using IHC. The expression of each protein was defined as high and low expression or positive and negative. The expression in individual patients was showed in Fig. 1. High expression of EGFR, HER2, HER3, HER4, VEGFR3, VEGF-C, EphA3, EphrinA1, p-Akt1, Akt1, beta-catenin and Wnt5a were 36, 55, 34, 34, 27, 61, 41, 52, 64, 49, 17 and 51%, respectively (Fig. 2). The expressions of all proteins were analyzed with post-operative recurrence including early and late recurrence in order to identify proteins that can be used for the prediction of tumor recurrence. In addition, the expression of beta-catenin was examined in the different cellular compartments, cytoplasm, membrane and nucleus. Positive expression of beta-catenin in cytoplasm, membrane and nucleus were 17, 8 and 2%, respectively. Among 12 protein kinases, the expression of EGFR, HER4, and EphA3 was significantly associated with early recurrence (*p* = 0.038: *p* = 0.033: *p* = 0.008; Table 2), while HER2 and p-Akt1 were significantly correlated with late recurrence (*p* = 0.035: *p* = 0.029; Table 2). In contrast, there was no correlation between HER3, VEGFR3, VEGF-C, EphrinA1, Akt1, beta-catenin, Wnt5a and post-operative recurrence (Table 2).

**Table 2:** The correlation of 12 protein kinases and post-operative recurrence of CCA patients

Protein kinases	Early recurrence	Late recurrence	No recurrence		*p* value	
	*n* = 31	*n* = 27	*n* = 132	Early vs No recurrence	Late vs No recurrence	Early vs Late recurrence
EGFR						
Low	14 (45)	19 (70)	88 (67)	**0.038**	0.824	0.067
High	17 (55)	8 (30)	44 (33)			
HER2						
Low	14 (45)	7 (26)	64 (49)	0.842	**0.035**	0.174
High	17 (55)	20 (74)	68 (51)			
HER3						
Low	22 (71)	18 (67)	86 (65)	0.674	1.000	0.781
High	9 (29)	9 (33)	46 (35)			
HER4						
Low	15 (48)	17 (63)	93 (71)	**0.033**	0.495	0.300
High	16 (52)	10 (37)	39 (29)			
VEGFR3						
Low	25 (81)	21 (78)	93 (71)	0.372	0.493	1.000
High	6 (19)	6 (22)	39 (29)			
VEGF-C						
Low	12 (39)	8 (30)	54 (41)	1.000	0.387	0.583
High	19 (61)	19 (70)	78 (59)			
EphA3						
Low	12 (39)	15 (56)	86 (65)	**0.008**	0.384	0.292
High	19 (61)	12 (44)	46 (35)			
EphrinA1						
Low	16 (52)	14 (52)	61 (46)	0.690	0.674	1.000
High	15 (48)	13 (48)	71 (54)			
p-Akt1						
Low	9 (29)	5 (19)	55 (42)	0.225	**0.029**	0.378
High	22 (71)	22 (81)	77 (58)			
Akt1						
Low	16 (52)	14 (52)	66 (50)	1.000	1.000	1.000
High	15 (48)	13 (48)	66 (50)			
Cytoplasmic beta-catenin						
Negative	27 (87)	22 (82)	108 (82)	0.603	1.000	0.720
Positive	4 (13)	5 (18)	24 (18)			
Membranous beta-catenin						
Negative	28 (90)	25 (93)	121 (92)	0.731	1.000	1.000
Positive	3 (10)	2 (7)	11 (8)			
Nuclear beta-catenin						
Negative	31 (100)	27 (100)	129 (98)	1.000	1.000	NA
Positive	0 (0)	0 (0)	3 (2)			
Wnt5a						
Low	16 (52)	9 (33)	68 (51)	1.000	0.095	0.192
High	15 (48)	18 (67)	64 (49)			

*EGFR* Epidermal growth factor receptor, *HER* Human epidermal growth factor receptor, *VEGFR3* Vascular endothelial growth factor receptor 3, *VEGF-C* Vascular endothelial growth factor-*C, EphA3* Erythropoietin-producing hepatocellular carcinoma receptor type-A3, *p-Akt1*: Phosphor-serine/threonine kinase 1, *Akt1* Serine/threonine kinase 1, *Wnt5a* Protein Wnt5a, *NA* Not applicable

**Fig. 3:**
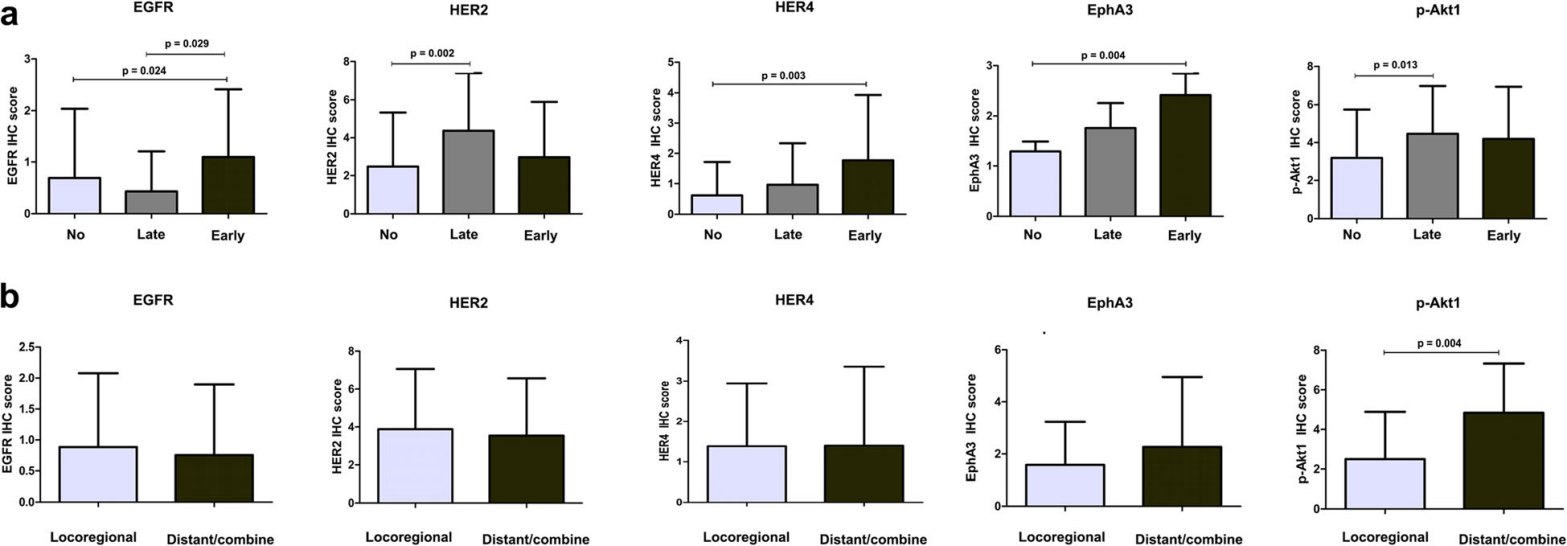
The expressing levels of protein kinases in patients with and without recurrence, and in the different recurrence location. a, The expressing levels of EGFR, HER2, HER4, EphA3 and p-Akt1 in different group of CCA patients which are no-recurrence (No, *n* = 132), late recurrence (Late, *n* = 27) and early recurrence (Early, *n* = 31). b, The expressing levels of EGFR, HER2, HER4, EphA3 and p-Akt1 in different recurrence location, locoregional (*n* = 13) and distant recurrence/combination between locoregional recurrence with distant recurrence (*n* = 45). *p*-value less than 0.05 was considered as statistical significance

The IHC scores of EGFR, HER2, HER4, EphA3 and p-Akt1 were also compared between patients with and without recurrence. The IHC score of EGFR was significantly different between patients with early recurrence compared with late or without recurrence (*p* = 0.029: *p* = 0.024; Fig. 3a). The IHC scores of HER2 and p-Akt1 were significantly higher in patients with late recurrence compared with no-recurrence (*p* = 0.002: *p* = 0.013; Fig. 3a), while IHC scores of HER4 and EphA3 were significantly higher in patients with early recurrence compared with no-recurrence (*p* = 0.003: *p* = 0.004; Fig. 3a). On the contrary, there was no difference between IHC scores of HER3, VEGFR3, VEGF-C, Ehprin-A1, Akt1, beta-catenin and Wnt5a (Fig. S1 and S2). The IHC scores of these proteins were also analyzed with recurrent location. The expressing level of p-Akt1 was significantly higher in the patients with distant recurrence/combination between locoregional recurrence with distant recurrence compared with locoregional recurrence (*p* = 0.004; Fig. 3b), while there was no statistical difference in EGFR, HER2, HER4 and EphA3 (Fig. 3b). The expression levels of EGFR, HER2, HER4, EphA3 and p-Akt1 were also analyzed with clinicopathological characteristics. Our finding only showed the sigfinicant correlation between expression of HER4 and lymph node metastasis (*p* = 0.045; Table 3).

**Table 3:** The correlation of EGFR, HER2, HER4, EphA3 and p-Akt1 expression with clinicopathological data

Variable	EGFR		*p*	HER2		*p*	HER4		*p*	EphA3		*p*	p-Akt1		*p*
	Low	High		Low	High		Low	High		Low	High		Low	High	
Sex															
Female	43 (36)	24 (35)	1.000	28 (33)	39 (37)	0.647	42 (34)	25 (39)	0.525	40 (35)	27 (35)	1.000	25 (36)	42 (35)	0.875
Male	78 (64)	45 (65)		57 (67)	66 (63)		83 (66)	40 (61)		73 (65)	50 (65)		44 (64)	79 (65)	
Age (year)															
< 61	60 (50)	32 (46)	0.763	37 (44)	55 (52)	0.245	61 (49)	31 (48)	1.000	55 (49)	36 (47)	0.768	38 (55)	54 (45)	0.177
≥ 61	61 (50)	37 (54)		48 (56)	50 (48)		64 (51)	34 (52)		57 (51)	41 (53)		31 (45)	67 (55)	
Tumor location															
Intrahepatic	61 (50)	44 (64)	0.095	48 (56)	57 (54)	0.771	64 (51)	41 (63)	0.127	63 (56)	42 (55)	0.883	35 (51)	70 (58)	0.366
Extrahepatic	60 (50)	25 (36)		37 (44)	48 (46)		61 (49)	24 (37)		50 (44)	35 (45)		34 (49)	51 (42)	
Histology															
Papillary	55 (46)	26 (38)	0.360	34 (40)	47 (45)	0.557	54 (43)	27 (42)	0.878	48 (42)	33 (43)	1.000	27 (39)	54 (45)	0.542
Others	66 (54)	43 (62)		51 (60)	58 (55)		71 (57)	38 (58)		65 (58)	44 (57)		42 (61)	67 (55)	
Primary tumor (T)															
I, II	75 (62)	34 (49)	0.096	50 (59)	59 (56)	0.769	77 (62)	32 (49)	0.122	68 (60)	41 (53)	0.372	44 (64)	65 (54)	0.222
III, IV	46 (38)	35 (51)		35 (41)	46 (44)		48 (38)	33 (51)		45 (40)	36 (47)		25 (36)	56 (46)	
Lymph nodes metastasis (N)															
No	67 (55)	38 (55)	1.000	46 (54)	59 (56)	0.883	76 (61)	29 (45)	**0.045**	66 (58)	39 (51)	0.302	37 (54)	68 (56)	0.763
Yes	54 (45)	31 (45)		39 (46)	46 (44)		49 (39)	36 (55)		47 (42)	38 (49)		32 (47)	53 (44)	
Distant metastasis (M)															
No	112 (93)	67 (97)	0.333	81 (95)	98 (93)	0.757	117 (94)	62 (95)	0.752	105 (93)	74 (96)	0.530	66 (96)	113 (93)	0.749
Yes	9 (7)	2 (3)		4 (5)	7 (7)		8 (6)	3 (5)		8 (7)	3 (4)		3 (4)	8 (7)	
TNM Stage															
I, II	53 (44)	23 (33)	0.169	35 (41)	41 (39)	0.768	56 (45)	20 (31)	0.064	47 (42)	29 (38)	0.652	31 (45)	45 (37)	0.356
III, IV	68 (56)	46 (67)		50 (59)	64 (61)		69 (55)	45 (69)		66 (58)	48 (62)		38 (55)	76 (63)	

*EGFR* Epidermal growth factor receptor, *HER* Human epidermal growth factor receptor, *EphA3* Erythropoietin-producing hepatocellular carcinoma receptor type-A3, *TNM* Size of primary tumor-node metastasis-distant metastasis

### The correlation of tumor maker level with post-operative recurrence

**Fig. 4:**
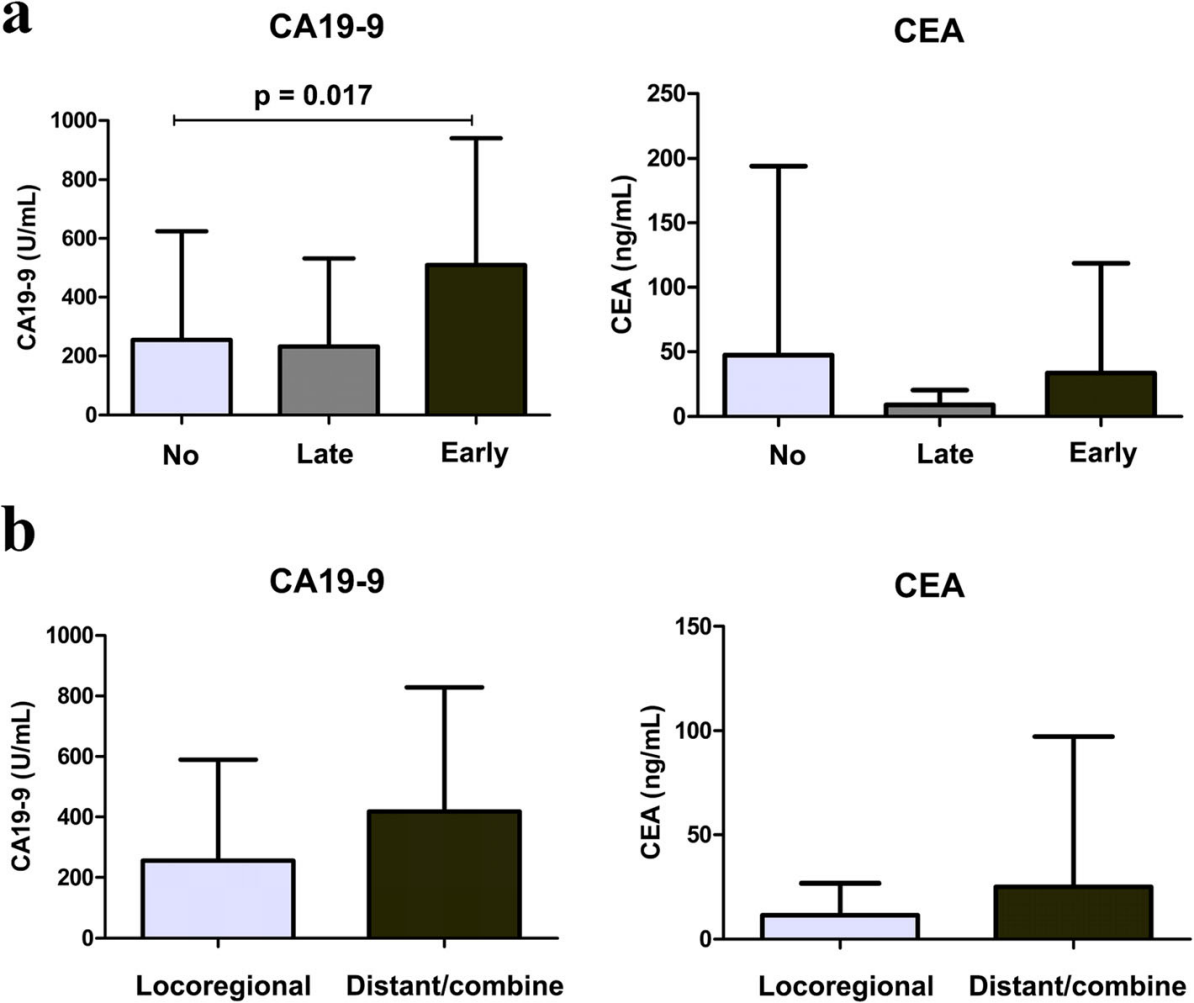
The levels of serum tumor markers in patients with and without recurrence, and in the different recurrence location. **a**, the levels of CA19–9 and CEA in different group of CCA patients which are no-recurrence (No, *n* = 81), late recurrence (Late, *n* = 19) and early recurrence (Early, *n* = 19). **b**, the levels of CA19–9 and CEA in different recurrence location, locoregional (*n* = 11) and distant recurrence/combination between locoregional recurrence with distant recurrence (*n* = 27). *p*-value less than 0.05 was considered as statistical significance

Since tumor markers were also used to monitor patients after treatment. Therefore, in the present study, CA19–9 and CEA levels were analyzed with tumor recurrence. The result revealed that the level of CA19–9 was significantly higher in early recurrence compared with no-recurrence (*p* = 0.017) (Fig. 4a), whereas there was no difference between CEA level in patients with and without recurrence (Fig. 4a). In addition, the levels of CA19–9 and CEA were also analyzed with recurrence location. All markers were likely to increase in distant recurrence/combination between locoregional recurrence with distant recurrence, compared with locoregional recurrence. However, there was no such statistically significant correlation in this study (Fig. 4b).

### The correlation of EGFR, HER2, HER4, EphA3, p-Akt1 and their prognostic significance

**Fig. 5:**
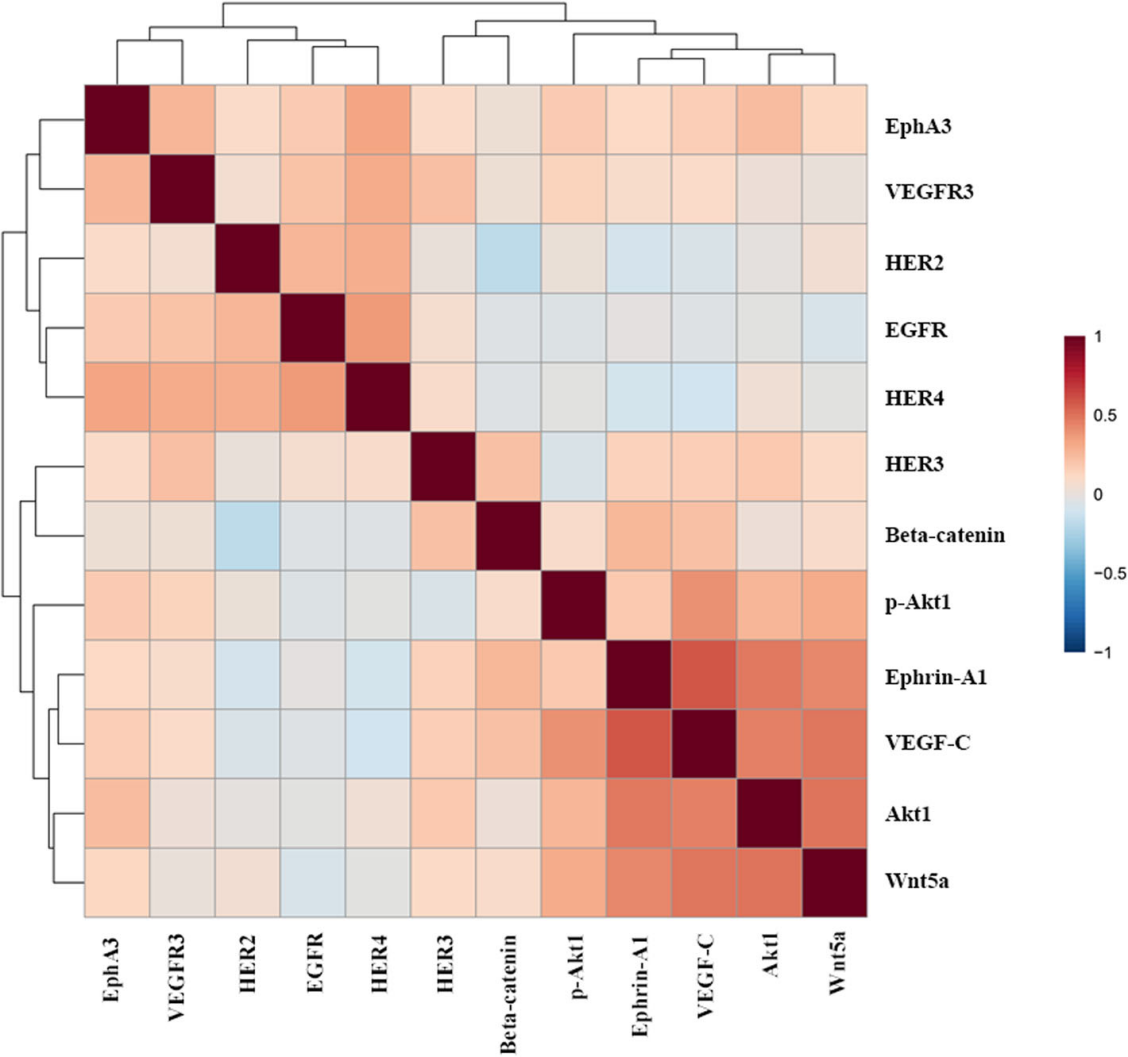
Correlogram of 12 protein kinase expression with clustering analysis performed in MetaboAnalyst 4.0. Data was analysed using Pearson correlation analysis. Correlation coefficient was indicated in each coloured cell on the map. The scale code shown on the right side (red and blue colours indicate positive and negative correlations, respectively.)

The correlations between EGFR, HER2, HER4, EphA3 and p-Akt1 were explored. The expression of EGFR was highly correlated with HER4 (*p* = 3.0 × 10^− 6^) and EphA3 (*p* = 1.8 × 10^− 4^). In addition, the expression of HER4 was also highly correlated with EphA3 (*p* = 1.2 × 10^− 8^). A positive correlation of HER2 with EGFR (*p* = 0.007) and HER4 (*p* = 0.013) was also found. On the other hand, there was no significant correlation between p-Akt1 and the other proteins (Table 4). Moreover, pearson correlation analysis on 12 protein kinases was also explored. There was a strong correlation between EGFR, HER4 and EphA3. (Fig. 5).

**Table 4:** Correlation coefficients between immunohistochemistry result of protein kinases

	HER2	HER4	EphA3	p-Akt1
EGFR Correlation coefficient	0.195	0.332	0.268	−0.044
*p*	**0.007**	**3.0 × 10**^**−6**^	**1.8 × 10**^**− 4**^	0.545
HER2 Correlation coefficient		0.180	0.096	0.025
*p*		**0.013**	0.186	0.735
HER4 Correlation coefficient			0.399	−0.078
*p*			**1.2 × 10**^**−8**^	0.283
EphA3 Correlation coefficient				− 0.023
*p*				0.752

*EGFR* Epidermal growth factor receptor, *HER* Human epidermal growth factor receptor, *EphA3*: Erythropoietin-producing hepatocellular carcinoma receptor type-A3, *p-Akt1* Phosphor-serine/threonine kinase 1

**Fig. 6:**
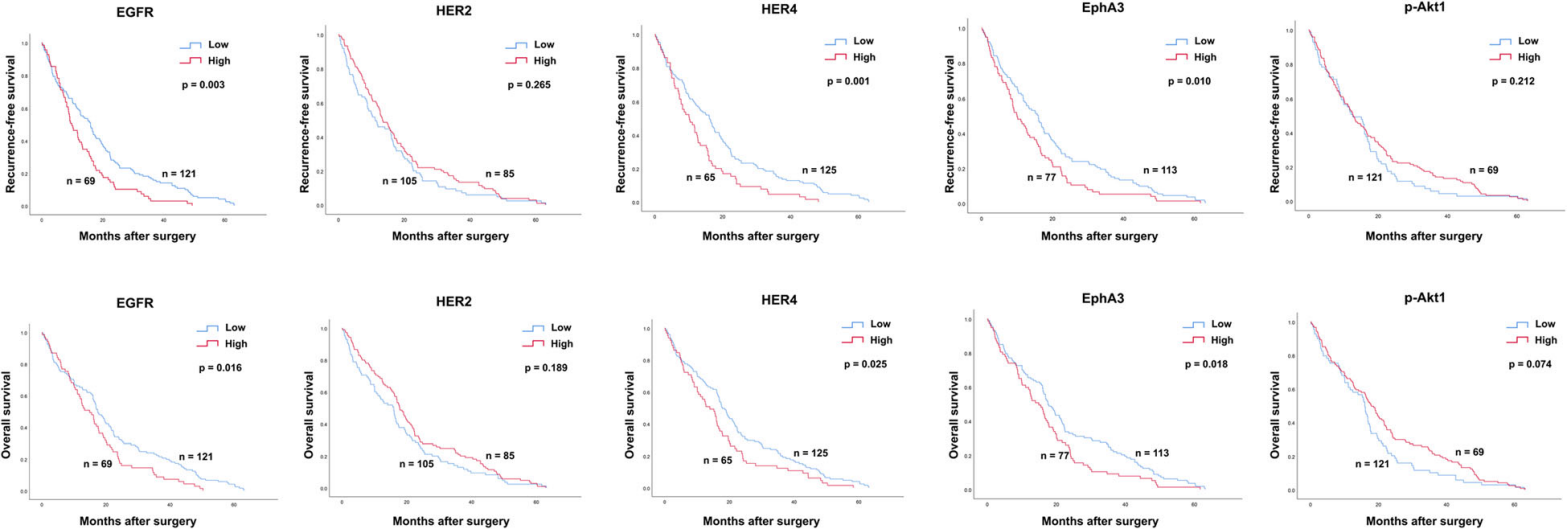
Kaplan-Meier analysis for RFS and OS according to EGFR, HER2, HER4, EphA3, and p-Akt1 expression. Upper panel, the prognostic significantly of EGFR, HER2, HER4, EphA3, and p-Akt1on RFS. Lower panel, the prognostic significantly of EGFR, HER2, HER4, EphA3, and p-Akt1on OS. *p*-value less than 0.05 was considered as statistical significance

**Fig. 7:**
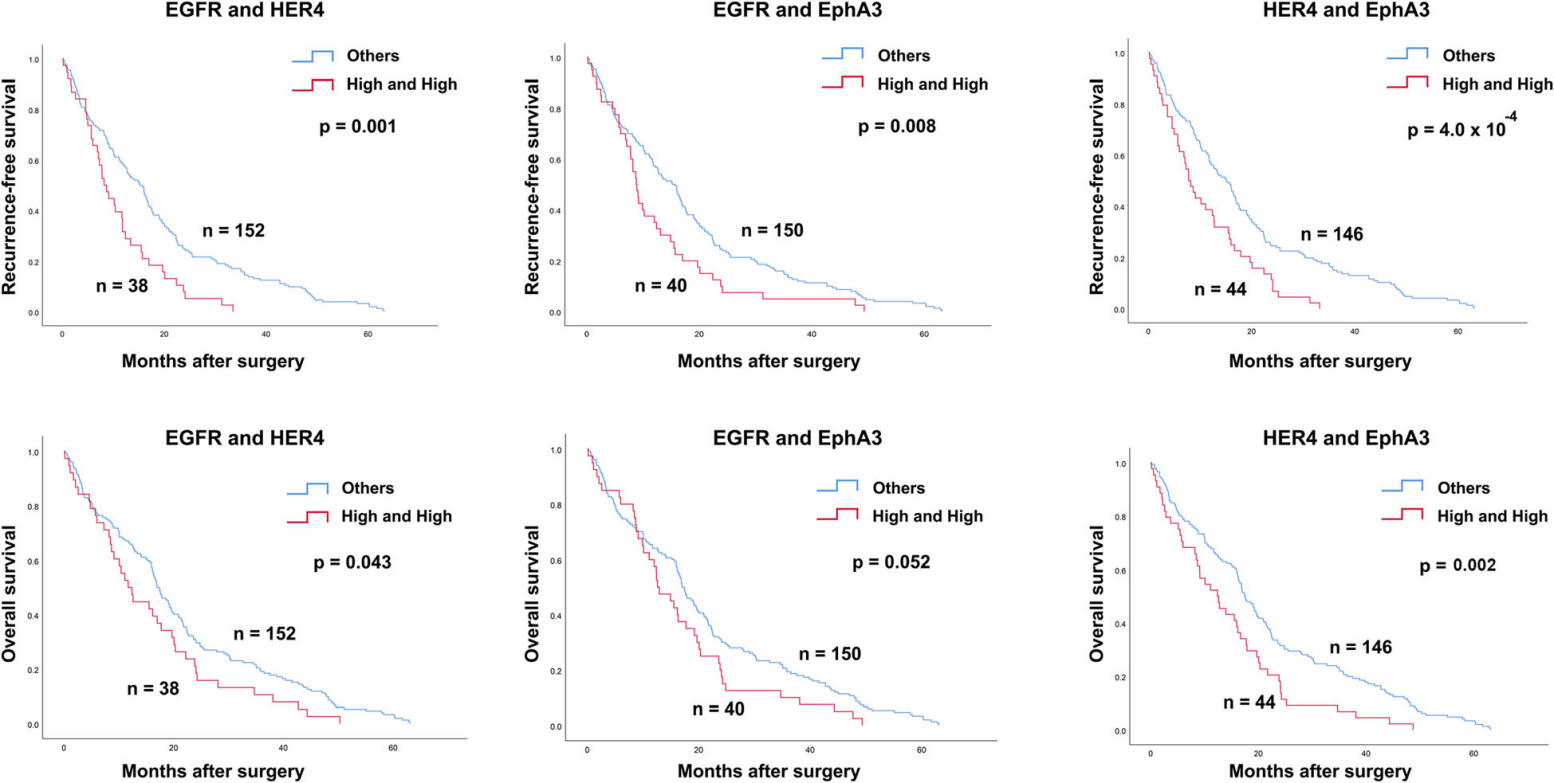
Kaplan-Meier analysis for RFS and OS according to the combined of two protein kinase expression. Upper and lower panels demonstrated the prognostic significantly of the combined of two protein kinase expression on RFS and OS, respectively. High and high represented the patients with high expression of two proteins, while other represented the patients with at least one protein low expression. *p*-value less than 0.05 was considered as statistical significance

**Fig. 8:**
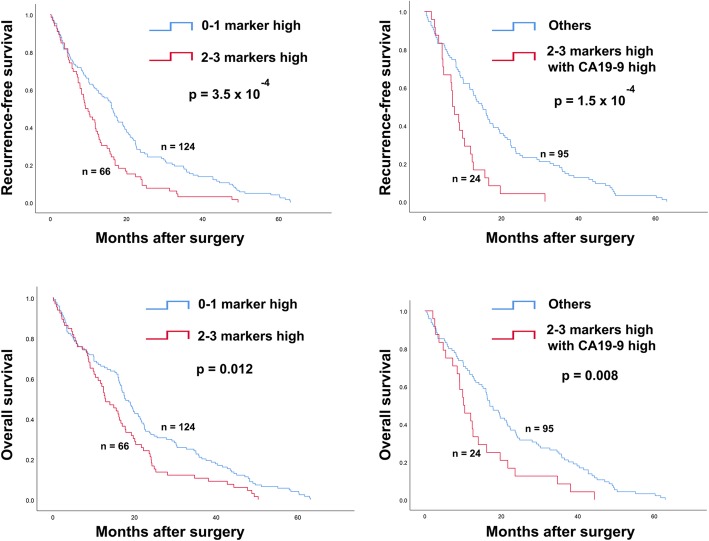
Kaplan-Meier analysis for RFS and OS according to the combined of three protein kinase expression (EGFR, HER4 and EphA3). Upper and lower panels demonstrated the prognostic significantly of the combined of three protein kinase expression or the combined of three protein kinase expression with CA19–9 level on RFS and OS, respectively. 0–1 marker high represented the patients with all markers low or one marker high, 2–3 markers high represented the patients with at least two markers high, others represented three groups of patients (0–1 marker high and CA19–9 low, 0–1 marker high and CA19–9 high or 2–3 markers high and CA19–9 low). *p*-value less than 0.05 was considered as statistical significance

The above results demonstrate that the expression of EGFR, HER2, HER4, EphA3 and p-Akt1 was significantly associated with post-operative recurrence. Thus, the expression of these proteins was then used to investigate their prognostic ability. Patients with high expression of EGFR, HER4 or EphA3 have shorter RFS (*p* = 0.003: *p* = 0.001: 0.010; Fig. 6) and OS (*p* = 0.016: *p* = 0.025: 0.018; Fig. 6), compared with those patients with low expression. However, there was no significance found in HER2 and p-Akt1 (Fig. 6). Because the expressing levels of EGFR, HER4 and EphA3 were highly correlated with each other, their expressing levels were also associated with patient prognosis. Therefore, the combination of these proteins was also analyzed with patient prognosis. High expression of the protein pairs, EGFR and HER4, EGFR and EphA3, and HER4 and EphA3 was significantly associated with shorter RFS (*p* = 0.001: *p* = 0.008: *p* = 4.0 × 10^− 4^; Fig. 7). High expression of EGFR and HER4, HER4 and EphA3 was also associated with a shorter OS (*p* = 0.043: *p* = 0.002; Fig. 7). In addition, patients who had high expression of two and three proteins were significantly associated with shorter RFS (*p* = 3.5 × 10^− 4^; Fig. 8) and OS (*p* = 0.012; Fig. 8). The level of tumor marker CA19–9 was also correlated with tumor relapse. Thus, the prognostic efficiency of the combination of protein kinases expression and tumor marker level was also explored. It was significantly associated with shorter RFS (*p* = 1.5 × 10^− 4^; Fig. 8) and OS (*p* = 0.008; Fig. 8).

### Independent prognostic value of EGFR, HER4 and EphA3

**Fig. 9:**
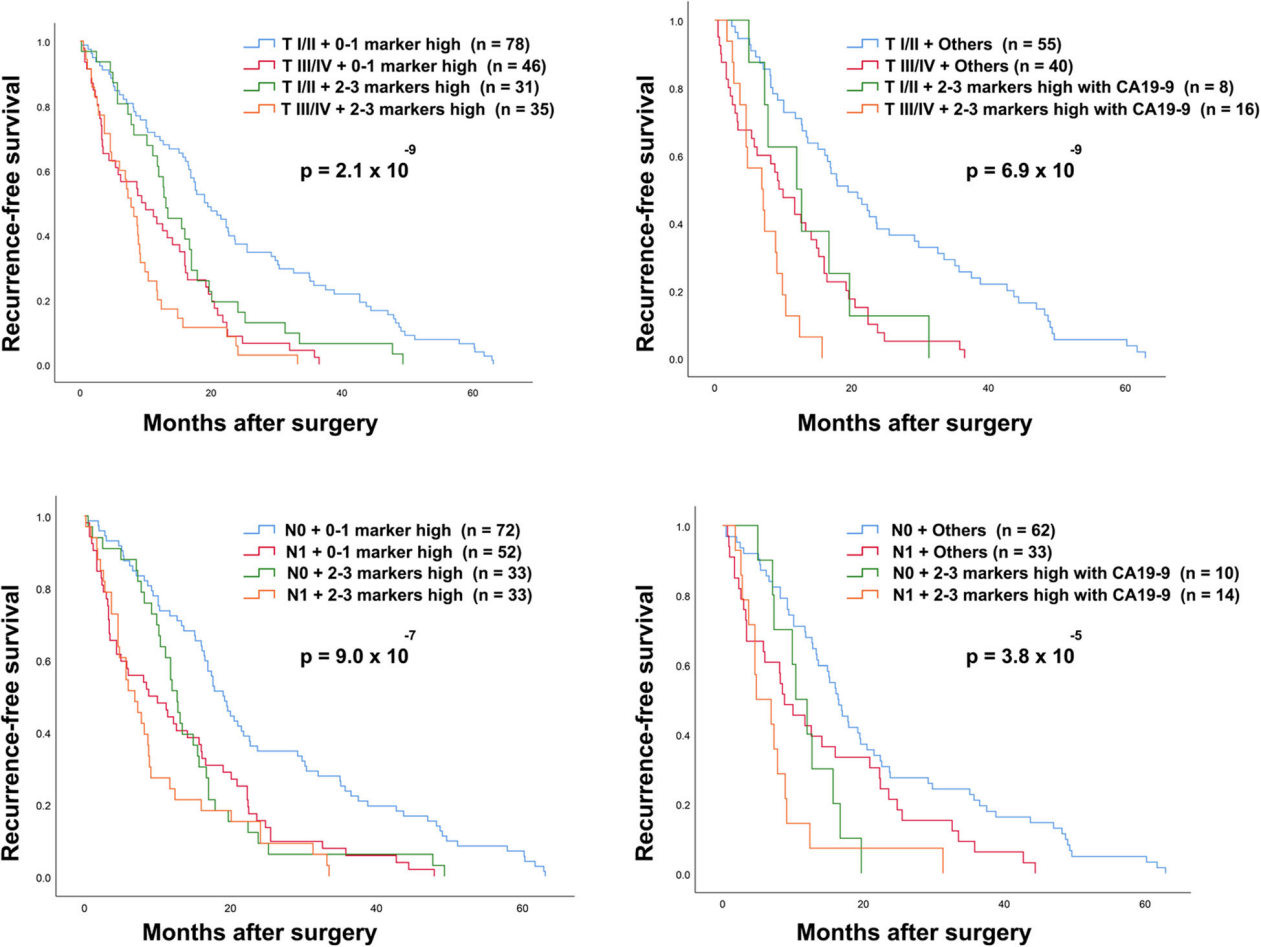
Kaplan-Meier analysis for RFS according to the combination of three protein kinase expression (EGFR, HER4 and EphA3) and clinicopathological features. 0–1 marker high represented the patients with all markers low or one marker high, 2–3 markers high represented the patients with at least two markers high, others represented three groups of patients (0–1 marker high and CA19–9 low, 0–1 marker high and CA19–9 high or 2–3 markers high and CA19–9 low). T represented the primary tumor stage, N represented the lymph node metastasis status (N0: no lymph node metastasis, N1: lymph node metastasis). *p*-value less than 0.05 was considered as statistical significance

In order to investigate whether EGFR, HER4 and EphA3 could be used as prognostic factors, independent of clinicopathological characteristics, Cox regression analysis was used. The univariate result for factors predicting the RFS and OS is shown in Table 5. Multivariate Cox regression for RFS and OS was analyzed using the different models that are summarized in Table 6 and Table 7. The result demonstrated that EGFR, HER4 and EphA3 were the independent prognostic factors for RFS (HR: 1.542; *p* = 0.006, HR:1.388; *p* = 0.042, HR: 1.469; *p* = 0.001; Table 6). EGFR and EphA4 were also independent prognostic factors for OS (HR: 1.450; *p* = 0.019, HR: 1.372; *p* = 0.040; Table 7). The combination of high expression of two and three markers or the high expression of two and three markers with high level of CA19–9 could be used to improve the predictive ability for RFS (HR: 1.528; *p* = 0008, HR: 2.080; *p* = 0.004; Table 6). Moreover, the patients were stratified more accurately when analyzed using the combintion of protein kinase expression and primary tumor (T) or lymph node metastasis (N) status. The patients with high stage T and high expression of two and three markers or high expression of two and three markers with high level of CA19–9 have shorter RFS, compared with other groups (*p* = 2.1 × 10^− 9^: *p* = 6.9 × 10^− 9^; Fig. 9). Similarly, patients with lymph node metastasis and high expression of two and three markers or high expression of two and three markers with a high level of CA19–9 have shorter RFS compared with other groups (*p* = 9.0 × 10^− 7^: *p* = 3.8 × 10^− 5^; Fig. 9).

**Table 5:** Univariate analysis of factors predicting recurrence-free and overall survival

Variable	Recurrence-free survival			Overall survival		
	HR	95% CI	*p*	HR	95% CI	*p*
Sex (Male vs Female)	1.159	0.856–1.569	0.340	1.132	0.837–1.532	0.422
Age (≥61 vs < 61)	1.144	0.858–1.525	0.359	1.302	0.975–1.737	0.073
Tumor location (Extra. Vs Intra.)	0.852	0.639–1.136	0.276	0.823	0.617–1.099	0.186
Histology (Others vs Papillary)	1.221	0.914–1.631	0.176	1.111	0.832–1.483	0.476
Primary tumor (T) (III/IV vs I/II)	2.474	1.811–3.378	**1.2 × 10**^**− 8**^	2.520	1.853–3.426	**3.7 × 10**^**−9**^
Lymph nodes metastasis (N) (Yes vs No)	1.968	1.459–2.655	**9.0 × 10**^**− 6**^	2.180	1.614–2.946	**3.8 × 10**^**−7**^
Distant metastasis (M) (Yes vs No)	1.783	0.967–3.289	0.064	2.093	1.133–3.869	**0.018**
TNM Stage (III/IV vs I/II)	2.387	1.738–3.278	**7.6 × 10**^**−8**^	2.564	1.865–3.525	**6.7 × 10**^**− 9**^
EGFR (High vs Low)	1.593	1.173–2.164	**0.003**	1.452	1.071–1.969	**0.016**
HER4 (High vs Low)	1.646	1.207–2.244	**0.002**	1.412	1.042–1.914	**0.026**
EphA3 (High vs Low)	1.465	1.091–1.966	**0.011**	1.424	1.060–1.913	**0.019**
Protein panel^a^ (2–3 markers high vs Others^b^)	1.746	1.281–2.380	**4.2 × 10**^**− 4**^	1.476	1.087–2.003	**0.013**
Combined of protein panel and CA19–9 level (2–3 markers high and CA19–9 high vs Others^c^)	2.435	1.512–3.921	**2.5 × 10**^**− 4**^	1.849	1.166–2.932	**0.009**

*TNM* Size of primary tumor-node metastasis-distant metastasis, *EGFR* Epidermal growth factor receptor, *HER* Human epidermal growth factor receptor, *EphA3* Erythropoietin-producing hepatocellular carcinoma receptor type-A3, Protein panel^a^: the expression of EGFR, HER4 and EphA, Others^b^: 0–1 marker high, Others^c^: three groups of patients (0–1 marker high and CA19–9 low, 0–1 marker high and CA19–9 high or 2–3 markers high and CA19–9 low)

**Table 6:** Multivariate analysis of factors predicting recurrence-free survival

Variable	Recurrence-free survival		
	HR	95% CI	*p*
Model A			
Primary tumor (T) (III/IV vs I/II)	2.058	1.364–3.104	**0.001**
Lymph nodes metastasis (N) (Yes vs No)	1.617	1.083–2.413	**0.019**
TNM Stage (III/IV vs I/II)	1.107	0.654–1.873	0.705
EGFR (High vs Low)	1.542	1.131–2.103	**0.006**
Model B			
Primary tumor (T) (III/IV vs I/II)	2.000	1.312–3.047	**0.001**
Lymph nodes metastasis (N) (Yes vs No)	1.459	0.963–2.211	0.075
TNM Stage (III/IV vs I/II)	1.158	0.668–2.009	0.601
HER4 (High vs Low)	1.388	1.011–1.906	**0.042**
Model C			
Primary tumor (T) (III/IV vs I/II)	1.977	1.296–3.017	**0.002**
Lymph nodes metastasis (N) (Yes vs No)	1.467	0.967–2.225	0.071
TNM Stage (III/IV vs I/II)	1.242	0.712–2.168	0.445
EphA3 (High vs Low)	1.469	1.091–1.977	**0.011**
Model D			
Primary tumor (T) (III/IV vs I/II)	1.918	1.255–2.931	**0.003**
Lymph nodes metastasis (N) (Yes vs No)	1.480	0.979–2.240	0.063
TNM Stage (III/IV vs I/II)	1.193	0.686–2.073	0.531
Protein panel^a^ (2–3 markers high vs Others^b^)	1.528	1.116–2.091	**0.008**
Model E			
Primary tumor (T) (III/IV vs I/II)	3.275	1.710–6.273	**3.5 × 10**^**−4**^
Lymph nodes metastasis (N) (Yes vs No)	1.749	1.017–3.009	**0.043**
TNM Stage (III/IV vs I/II)	0.660	0.292–1.496	0.320
Combined of protein panel and CA19–9 level (2–3 markers high and CA19–9 high vs Others^c^)	2.080	1.270–3.405	**0.004**

*TNM* Size of primary tumor-node metastasis-distant metastasis, *EGFR* Epidermal growth factor receptor, *HER* Human epidermal growth factor receptor, *EphA3* Erythropoietin-producing hepatocellular carcinoma receptor type-A3, Protein panel^a^: the expression of EGFR, HER4 and EphA3, Protein panel^a^: the expression of EGFR, HER4 and EphA, Others^b^: 0–1 marker high, Others^c^: three groups of patients (0–1 marker high and CA19–9 low, 0–1 marker high and CA19–9 high or 2–3 markers high and CA19–9 low)

**Table 7:** Multivariate analysis of factors predicting overall survival

Variable	Overall survival		
	HR	95% CI	*p*
**Model A**			
Primary tumor (T) (III/IV vs I/II)	2.056	1.335–3.166	**0.001**
Lymph nodes metastasis (N) (Yes vs No)	1.750	1.152–2.660	**0.009**
Distant metastasis (M) (Yes vs No)	1.146	0.599–2.192	0.680
TNM Stage (III/IV vs I/II)	1.100	0.632–1.915	0.735
EGFR (High vs Low)	1.450	1.063–1.978	**0.019**
**Model B**			
Primary tumor (T) (III/IV vs I/II)	1.991	1.286–3.084	**0.002**
Lymph nodes metastasis (N) (Yes vs No)	1.642	1.067–2.526	**0.024**
Distant metastasis (M) (Yes vs No)	1.106	0.577–2.119	0.761
TNM Stage (III/IV vs I/II)	1.149	0.650–2.032	0.632
HER4 (High vs Low)	1.198	0.875–1.641	0.259
**Model C**			
Primary tumor (T) (III/IV vs I/II)	1.931	1.243–3.002	**0.003**
Lymph nodes metastasis (N) (Yes vs No)	1.594	1.026–2.446	**0.038**
Distant metastasis (M) (Yes vs No)	1.159	0.604–2.223	0.657
TNM Stage (III/IV vs I/II)	1.224	0.686–2.182	0.494
EphA3 (High vs Low)	1.372	1.014–1.855	**0.040**
**Model D**			
Primary tumor (T) (III/IV vs I/II)	1.925	1.237–2.995	**0.004**
Lymph nodes metastasis (N) (Yes vs No)	1.631	1.060–2.509	**0.026**
Distant metastasis (M) (Yes vs No)	1.134	0.591–2.176	0.706
TNM Stage (III/IV vs I/II)	1.178	0.665–2.089	0.574
Protein panel^a^ (2–3 markers high vs Others^b^)	1.269	0.926–1.738	0.138
**Model E**			
Primary tumor (T) (III/IV vs I/II)	4.006	2.033–7.893	**6.0 × 10**^**−5**^
Lymph nodes metastasis (N) (Yes vs No)	2.055	1.160–3.640	**0.014**
Distant metastasis (M) (Yes vs No)	0.781	0.323–1.888	0.583
TNM Stage (III/IV vs I/II)	0.604	0.262–1.389	0.235
Combined of protein panel and CA19–9 level (2–3 markers high and CA19–9 high vs Others^c^)	1.490	0.922–2.406	0.103

*TNM*: Size of primary tumor-node metastasis-distant metastasis, *EGFR* Epidermal growth factor receptor, *HER* Human epidermal growth factor receptor, *EphA3* Erythropoietin-producing hepatocellular carcinoma receptor type-A3, Protein panel^a^: the expression of EGFR, HER4 and EphA3, Protein panel^a^: the expression of EGFR, HER4 and EphA, Others^b^: 0–1 marker high, Others^c^: three groups of patients (0–1 marker high and CA19–9 low, 0–1 marker high and CA19–9 high or 2–3 markers high and CA19–9 low)

## Discussion

The treatment of CCA is challenging since most of the patients were diagnosed when the disease reaches advance stage. Surgical resection is, nowadays, only curative method for CCA treatment, and usually suitable for patients in early stage [17]. Some patients also receive adjuvant chemotherapy or radiotherapy for improving patient’s survival [18]. However, many patients developed tumor recurrence even with complete resection [4]. The recurrence rates of CCA were shown differently depending on the studies of which the rates were reported between 29 and 80% [4, 5, 19]. In this study, the recurrence rate was 31%. Recurrence was found in both locoregional and distant recurrence, and most of patients developed distant recurrence. Among the patients with distant recurrence, the highest recurrence rate was found in the liver followed by peritoneum. Our finding is consistent with the previous study exhibiting that the common site of recurrence was distant recurrence with accounting for 54.3%. Among them, liver (43%) and peritoneum (26.3%) were the common sites of recurrence, resulting from the hematogenous spread of tumor [20].

Since tumor recurrence affects the outcome of cancer patients, there are several studies thus focused on an identification of marker which can predict tumor recurrence. Clinical features of patient were previously reported as predictor for CCA recurrence including tumor size, lymph node metastasis [21, 22], lymphatic infiltration, bile duct invasion, intrahepatic metastasis [23]. This is consistent with our study, demonstrating the primary tumor together with the presence of lymph node metastasis that effectively indicate the prognostic factor for CCA recurrence. However, biomarkers are still needed because the absence or presence of these markers can be used to predict the outcome of patients and could be useful for targeted therapy to prevent the worse outcome of patients.

EGFR family is known as protein kinases which are involved in many cellular processes via the activation of various downstream pathways [24]. The EGFR family consists of four members: EGFR (HER1), HER2, HER3 and HER4. The overexpression of EGFR is more commonly found in the primary tumor stages III/IV than I/II [25], and has been previously identified as a predictor of tumor recurrence [26–28]. Moreover, knockdown of EGFR expression could reduce the colony formation, migration and proliferation of colorectal cancer (CRC) [27]. These suggest that elevated EGFR is associated with tumor aggressiveness. Our study confirms the predictive value of this protein, even though a significant correlation between EGFR and clinicopathological features was not observed. In the EGFR family, the function and predictive ability of HER4 is poorly understood, compared with other members. HER4 is known because of its role on cancer progression. High expression of HER4 was also correlated with in triple negative breast cancer recurrence [29]. We found that HER4 expression was correlated with CCA recurrence and associated with shorter RFS and OS. Our finding is consistent with the previous report which explored the mechanism of tumor relapse after treatment. They found that activation of ligand-dependent HER4 signal plays an important role in tumor relapse via induce chemoresistance [30]. Eph receptor is another receptor tyrosine kinase which has been studied in CCA. The interaction of Eph receptor and ephrin ligand associated with the modification of actin cytoskeleton, adhesion, as well as cell shape [31]. In addition, upregulation of EhpA3 has also been reported to associate with tumor metastasis and recurrence in gastric cancer [32]. The previous study, exploring the mechanism underlying Eph receptor induce cancer recurrence demonstrated that co-expression of EphA2 and EphA3 led to the high clonogenicity and tumorigenic potential in recurrence of glioblastoma which has been shown in both in vitro and in vivo models. Moreover, co-targeting of EphA2 and EphA3 could also reduce clonogenic ability and tumorigenesis [33]. This may be confirmed by our study which also showed the association between EhpA3 and CCA recurrence.

Serum tumor marker, the well-established marker for monitoring tumor, is normally used for monitoring the progression of several types of cancer. In CCA, CA19–9 and CEA are the most widely used to monitor the outcome of CCA patients [34], even though it is not specific to only CCA. In this study, we also analyzed the association between CA19–9 and CEA levels with tumor recurrence. Among them, CA19–9 level was found higher in patients with recurrence than those without recurrence. Similarly, to another study which reported that CA19–9, it was shown as the independent prognosis factor for CCA recurrence [7].

A panel of three proteins (PI3K-p85α, EGFR and p53) has been identified as the independent prognostic factor in esophageal squamous cell carcinoma (ESCC), and the combination of a three protein panel with clinicopathological parameters, lymph node metastasis status and pathologic stage could classify patients into the different prognostic groups [25]. In our study, the combination of two and three proteins (EGFR, HER4 and EphA3) or the combination of these proteins with CA19–9 was an independent prognostic factor for tumor recurrence. Moreover, the patients were classified more accurately when analyzed using the combination of protein kinase expression and primary tumor (T) or lymph node metastasis (N) status. This may be beneficial for CCA patients to predict their outcome after surgical treatment and may be used as a guideline for clinical intervention in order to improve patient survival.

## Conclusion

Our results demonstrate that the elevated expression of EGFR, HER4, and EphA3 is correlated with OV-associated CCA recurrence. Moreover, the panel of high expression of EGFR, HER4, and EphA3 can be used as a prognostic factor for CCA recurrence, especially when combined with CA19–9 or clinicopathological features, primary tumor (T) or lymph node metastasis (N) status.

## Supplementary information

**Additional file 1: Fig. S1** The expressing levels of protein kinases in patients with and without recurrence, and in the different recurrence location. **a**, The expressing levels of protein kinases in different group of CCA patients which are no-recurrence (No, *n* = 132), late recurrence (Late, *n* = 27) and early recurrence (Early, *n* = 31). **b**, The expressing levels of protein kinases in different recurrence location, locoregional (*n* = 13) and distant recurrence/combination between locoregional recurrence with distant recurrence (*n* = 45). *p*-value less than 0.05 was considered as statistical significance.

**Additional file 2: Fig. S2** The expressing levels of beta-catenin in patients with and without recurrence in the different cellular compartments, cytoplasm, membrane and nucleus. The expressing levels of beta-catenin in different group of CCA patients which are no-recurrence (No, *n* = 132), late recurrence (Late, *n* = 27) and early recurrence (Early, *n* = 31). *p*-value less than 0.05 was considered as statistical significance.

**Additional file 3: Table S1** Patients characteristics.

## Data Availability

The datasets generated during and/or analyzed during the current study are available from the corresponding author on reasonable request.

## Abbreviations

Akt: Serine/threonine kinase or protein kinase B
CA19–9: Carbohydrate antigen 19-9
CCA: Cholangiocarcinoma
CEA: Carcinoembryonic antigen
EGFR: Epidermal growth factor receptor
Eph: Erythropoietin-producing hepatocellular carcinoma receptor
HER: Human epidermal growth factor receptor
OV: *Opisthorchis viverrini*
VEGF-C: Vascular endothelial growth factor C
VEGFR3: Vascular endothelial growth factor receptor 3
