# Supplementary figures and images for: A panel of protein kinase high expression is associated with postoperative recurrence in cholangiocarcinoma
Source: BMC Cancer. 2020 Feb 24;20:154. doi: 10.1186/s12885-020-6655-4 (PMC7041295; doi:10.1186/s12885-020-6655-4)

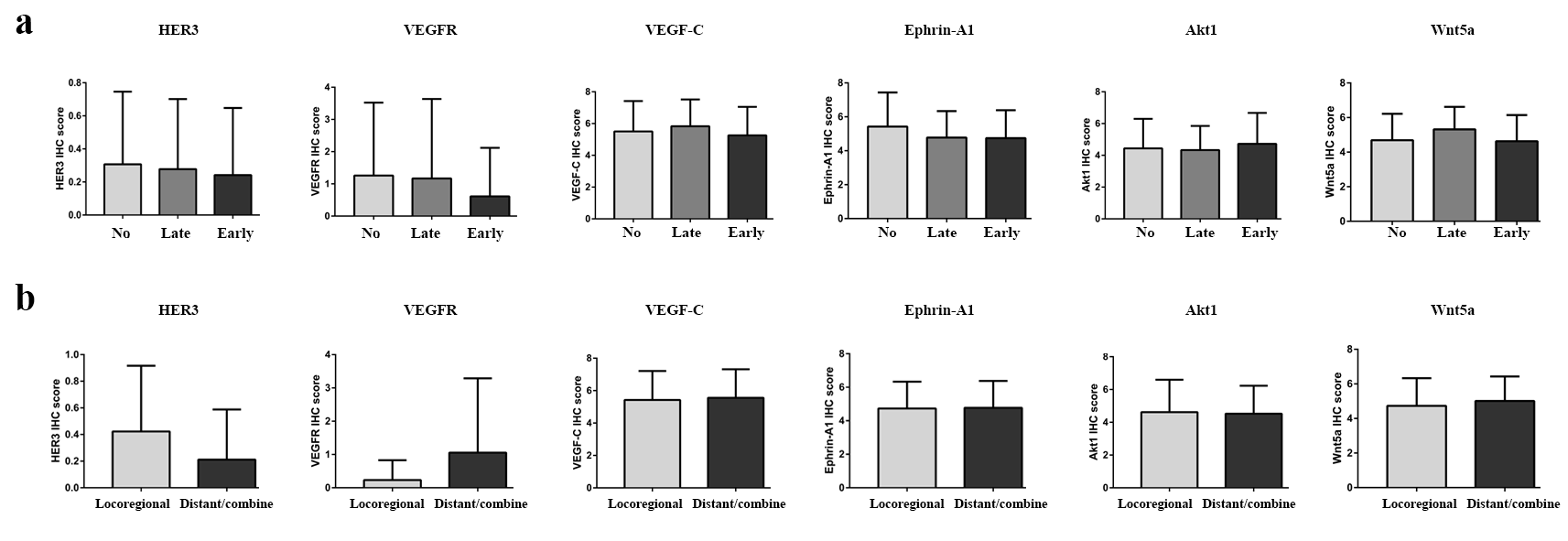

Supplement: Supplementary file 1 — Additional file 1: Fig. S1 The expressing levels of protein kinases in patients with and without recurrence, and in the different recurrence location. a, The expressing levels of protein kinases in different group of CCA patients which are no-recurrence (No, n = 132), late recurrence (Late, n = 27) and early recurrence (Early, n = 31). b, The expressing levels of protein kinases in different recurrence location, locoregional (n = 13) and distant recurrence/combination between locoregional recurrence with distant recurrence (n = 45). p-value less than 0.05 was considered as statistical significance. [file 12885_2020_6655_MOESM1_ESM.tif]

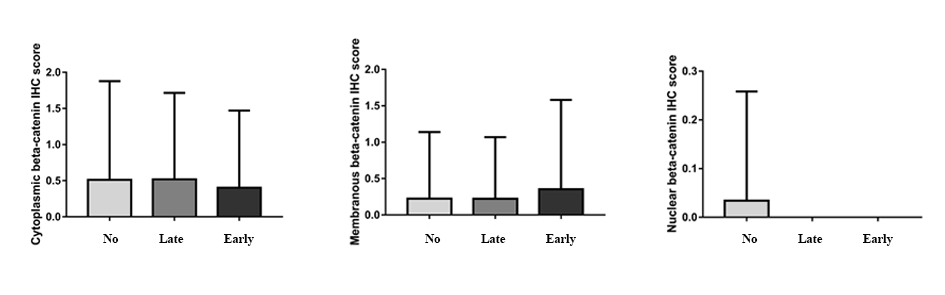

Supplement: Supplementary file 2 — Additional file 2: Fig. S2 The expressing levels of beta-catenin in patients with and without recurrence in the different cellular compartments, cytoplasm, membrane and nucleus. The expressing levels of beta-catenin in different group of CCA patients which are no-recurrence (No, n = 132), late recurrence (Late, n = 27) and early recurrence (Early, n = 31). p-value less than 0.05 was considered as statistical significance. [file 12885_2020_6655_MOESM2_ESM.tif]
