# Supplementary material for: A panel of protein kinase high expression is associated with postoperative recurrence in cholangiocarcinoma
Source: BMC Cancer. 2020 Feb 24;20:154. doi: 10.1186/s12885-020-6655-4 (PMC7041295; doi:10.1186/s12885-020-6655-4)
Supplement: Supplementary file 3 — Additional file 3: Table S1 Patients characteristics. [file 12885_2020_6655_MOESM3_ESM.docx]

**Table S1** Patients characteristics

| **Characteristics** | **Number of CCA patients (%)** |
| --- | --- |
| Sex  Female  Male | 67 (35)  123 (65) |
| Age (year)  < 61  ≥ 61 | 92 (48)  98 (52) |
| Tumor location  Intrahepatic  Extrahepatic | 105 (55)  85 (45) |
| Histology  Papillary  Others | 81 (43)  109 (57) |
| Primary tumor (T)  I, II  III, IV | 109 (57)  81 (43) |
| Lymph nodes metastasis (N)  No  Yes | 105 (55)  85 (45) |
| Distant metastasis (M)  No  Yes | 179 (94)  11 (6) |
| TNM Stage  I, II  III, IV | 76 (40)  114 (60) |
| Recurrence  No  Yes | 132 (69)  58 (31) |
| TNM: size of primary tumor-node metastasis-distant metastasis. | |
